# Synthesis and Evaluation of Benzophenone-N-ethyl Morpholine Ethers as Anti-inflammatory Agents

**Published:** 2010-03

**Authors:** Shaukath A. Khanum, Bushra A. Begum, V. Girish, Noor Fatima Khanum

**Affiliations:** 1 *Department of Chemistry, Yuvaraja college, University of Mysore, Mysore Karnataka, India;*; 2 *Department of Chemistry, D’Banumaih’s P U Science College, Mysore, Karnataka, India;*; 3 *Department of Food Science and Nutrition, Maharani’s Science College for Women, Mysore, Karnataka, India*

**Keywords:** morpholines, anti-inflammatory, ulcerogenic, cyclooxygenase, acute toxicity

## Abstract

The synthesis of hydroxy benzophenones and benzophenone-N-ethyl morpholine ethers and the results of anti-inflammatory activity *in vivo* are described. The structures of the compounds were elucidated by IR, ^1^H-NMR, mass spectroscopy and the elementary analysis. The anti-inflammatory activity of the synthesized compounds were determined by carrageenan-induced hind paw oedema test in rats. Most of the tested compounds exhibited anti-inflammatory activity and some of them were more active than standard drugs. In addition ulcerogenic and cyclooxygenase activities are also described.

## INTRODUCTION

Inflammation is a response of a tissue to injury, often injury caused by invading parasites (1, 2). The potent mediators of inflammation are derivatives of arachidonic acid a 20-carbon unsaturated fatty acid produced from membrane phospholipids. The principal pathways of arachidonic acid metabolism are the 5-lipoxygenase pathway, which produces a collection of leukotrienes and the cyclooxygenase (COX) pathway, which produces prostaglandin (3–5). Pharmacological inhibition of COX can provide relief from the symptoms of inflammation and pain, this is the method of action of non-steroidal anti-inflammatory drugs, such as the well known aspirin, phenyl butazone, indomethacin, ibuprofen etc. The names “prostaglandin synthase” and “prostaglandin synthetase” are still sometimes used to refer to the COX enzyme (6, 7). Recent studies have shown that COX exists in two isoforms COX-1 and COX-2. Both COX are constitutively expressed in most tissues, but COX-2, in contrast to COX-1, is the mitogen inducible isoform. The inducing stimuli for COX-2 include pro-inflammatory cytokines and growth factors, implying a role for COX-2 in both inflammation and control of cell growth (8–10). COX isoforms are almost identical in structure but have important differences in substrate and inhibitor selectivity and in their intracellular locations (11).

The pharmacological effect of morpholine compounds in different biological fields is of great importance for researchers and investigators. Chemically synthesized morpholino compounds are tested for its analgesic and inflammatory activities in albino rat (12, 13). These findings reflect a new fact of pharmacological action of these compounds like inhibition of arachidonic acid pathway *in vivo* (14). In addition, chloro (15) and bromo (16) substituted phenyl. morpholine analogues have shown good anti-inflammatory activity. Beside anti-inflammatory activity, morpholine analogues exhibit anti-cancer (17) and anti-microbial (18) activity.

The precursor hydroxy benzophenones for the synthesis of the title compounds are achieved from natural products (19) as well as by synthetic methods (20). The great importance of these substances is essentially due to the diverse biological and chemical properties they acquire. Benzophenone analogues possess a high analgesic (21) efficacy and also endowed with anti-inflammatory property (22). Several attempts to derive COX selective inhibitors from the non-selective NSAIDs like benzophenone analogue (23) has been published and is indicated in the treatment of rheumatoid arthritis, ankylosing spondylitis and osteoarthritis. In addition, halo amino benzophenones have shown excellent anti-inflammatory activity (22). The literature investigation revealed that no efforts were aimed towards the designing of N-ethyl morpholine moiety integrated with benzophenone framework to verify the importance of this moiety on the pharmacological activity. In view of this information and in our search for new molecules with anti-inflammatory activity (24, 26), it was considered valuable to synthesize benzophenone-N-ethyl morpholine ethers (5a-j) as anti-inflammatory agents for a rational study of the structure-activity relationships.

## METHODS

IR spectra were recorded in Nujol on FT-IR Shimadzu 8300 spectrophotometer, ^1^H NMR spectra were recorded on a Bruker 300 MHz NMR spectrophotometer in CDCl_3_ and chemical shift were recorded in parts per million down field from tetramethylsilane. Mass spectra were obtained with a VG70-70H spectrophotometer and important fragments are given with the relative intensities in the brackets. Elemental analysis results are within 0.4% of the calculated value.

**Figure 1:**
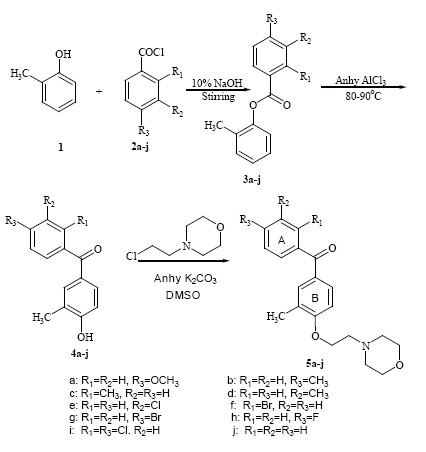
Synthesis of Benzophenone-N-ethyl Morpholine Ethers.

Substituted 2-methylphenyl benzoates (3a-j) were synthesized by stirring 2-methyl phenol (1, 1 equivalent) with corresponding benzoyl chlorides (2a-j, 1 equivalent) for 30 minutes in alkaline medium using 10% sodium hydroxide solution with excellent yield (85–90%). Substituted hydroxy benzophenones (4a-j) were synthesized by refluxing a mixture of 3a-j (1 equivalent) and anhydrous aluminium chloride (1.5 equivalent) for 20 minutes (24, 25) with 80–83% yield. Condensation of 4a-j (1 equivalent) with 4-(2-chloroethyl) morpholine hydrochloride (1 equivalent) for 3 hours in presence of anhydrous potassium carbonate (1.5 equivalent) and dimethyl sulphoxide furnished substituted benzophenone-N-ethyl morpholine ethers (5a-j, Fig. 1) with 70–75% yield. The compounds 5a-j (27) were characterized by IR, ^1^H NMR and mass spectrophotometer.

The animal experiments with Albino rats were carried out at Department of studies in Applied Botany and Biotechnology, University of Mysore, Manasagangotri, Mysore and permission for conducting these animal experiments was obtained from institutional Animals Ethics Committee.

### Anti-Inflammatory activity

Anti-inflammatory activity was performed by paw oedema inhibition test adopting Winter *et al* (28) method. Groups of five rats (body weight 200–230 g), were given a dose of a test compound. After 30 min, 0.1 ml of 1% carrageenan suspension in 0.9% sodium chloride solution was injected subcutaneously, into planter aponeurosis of the hind paw and the paw volume was measured by a water plethysmometer socrel and then measured again after a time span of 3 h. The mean increase of paw volume at each time interval was compared with that of control group (five rats treated with carrageenan, but not with test compounds) at the same time intervals. The percentage inhibition values were calculated using the formula:

$$\%\;\text{antiinflammatory}\;\text{activity}\;=1-{\text{V}}_{\text{t}}/{\text{V}}_{\text{c}}\times 100$$

where V_t_ and V_c_ are the mean relative changes in the volume of paw oedema in the test compounds and control respectively.

### Ulcerogenic activity

Groups of 10 rats (body weight 200–230 g), fasted for 24 h. were treated with an oral dose of test compound, except control group. All animals were sacrificed 5 h after the completion of dosing. With the aid of a microscope the stomach and small intestine of the rats were examined to find incidence of hyperemia, shedding of epithelium, petechial, frank hemorrhages and erosion or discrete ulceration with or without perforation. The presence of any of these criteria was considered to be an evidence of ulcerogenic activity (29).

### Acute toxicity study

ALD_50_ of the compounds was determined in albino rats (body weight 200–230 g). The test compounds were injected intra peritoneally at different dose levels in groups of 10 animals. After 24 h of drug administration, percent mortality in each group was observed from the data obtained ALD_50_ was calculated by adopting previous method (30).

### Cyclooxygenase activity

To search plausible mechanism of the compounds, *in vitro* test on microsomal fraction of mucosal preparation of rabbit distal colon was carried out using previous procedure (31). About 2–3 g of stripped, colonic mucosa was minced and homogenized in 3 volumes of Tris buffer 0.1M, pH 8.0 and the homogenized was centrifuged. The precipitate was suspended in Tris buffer 0.1 M, pH 8.0, and recentrifuged. For enzyme assay cyclooxygenase activity, the microsomal pellet was used immediately. By measuring the rate of conversion of arachidonic acid to PGE_2_, cyclooxygenase activity was assayed. About 50 ml of microsomal fractions was incubated with test agents for 10 min at 37°C in 30 μl tris-HCl, pH 8.0 containing 2 mM reduced glutathione, 5 mM L-tryptophan, 1 μM hematin. The substrate 20 μM arachidonic acid with tracer amount of [1-^14^C] arachidonic acid was then added and the reaction proceeded for 5 min at 37°C. The reaction was stopped by addition of 0.2 ml of ether/methanol/citric acid 0.2 M (30:4:1 v/v), which was precooled at −25°C PGE_2_, was extracted twice into the same mixture. The solvent was removed under nitrogen stream and radiolabelled arachidonic acid was separated and from this radiolabelled PGE_3_ was separated by HPLC with 2 nmol unlabelled PGE_2_ as an interval standard. PG chromatographic profile was obtained by isocratic elution with 150 mM orthophosphoric acid in water, pH 3.5, containing 30% acetonitrile, a flow rate of 1 ml/min monitoring the UV absorption at 214 nm. Radioactivity that co-eluted with authentic PGE_2_ was quantified by liquid scintillation spectrometry. Test samples were compared to paired control incubations. The percentage of inhibition was calculated as follows:

cpm control−cpm test/cpm control×100

## RESULTS

The attribute feature of the title compounds 5a-j is owing to the presence of benzophenone and morpholine moieties. Most of the title compounds exhibited excellent anti-inflammatory activity in the range 29.5 to 58.7% at a dose of 40 mg/kg po. In 5a-j series the compound 5f elicited maximum inhibition of oedema (58.7%). whereas compound 5d elicited minimum inhibition of oedema (29.5%). Compound 5a has shown second highest anti-inflammatory activity (55.5%). Besides compounds 5h (48.9%) and 5b (48.2%) have shown almost same anti-inflammatory activity. On the other hand compound 5e (49.9%) has exhibited anti-inflammatory activity little higher than compounds 5h and 5b. Nevertheless compound 5i (46.4%) has exhibited slightly more anti-inflammatory activity compared to the compound 5g (45.5%). The remaining compounds 5c and 5j have exhibited inhibition in oedema as 39.4 and 35.5% respectively.

Compounds 5a, 5b, 5e, 5f and 5h were studied in detail at three graded doses and have shown dose dependent activity. Anti-inflammatory activity of compounds 5a-j and their comparison with standard drugs, aspirin and phenylbutazone are given in table 1.

**Table 1:** Antiinflammatroy, ulcerogenic, cyclooxygenase and toxicity data of compounds 5a-j

Compd.	Dose (mg/kg po)	Anti-inflammatory activity % oedema inhibition relative to control	Dose (mg/kg po)	Ulcerogenic	activity	Cyclooxygenase activity assay inhibitory action of some selected compounds % inhibition 10 μM	ED_50_ (mg/kg po)	ALD_50_ (mg/kg po)
				% of animal with hyperemia	% of animal with ulcer			

5a	20	35.3	100	30	10	65	51.2	>1000
	40	55.5	200	60	10			
	80	84.4	400	90	12			
5b	20	17.4	100	50	05	70	77.2	>1000
	40	48.2	200	70	10			
	80	64.1	400	90	15			
5c	20	20.3	100	70	10	40	62.5	>1000
	40	39.4	200	90	20			
	80	62.2	400	100	40			
5d	20	14.1	100	40	10	63	78.3	>1000
	40	29.5	200	60	20			
	80	55.3	400	100	40			
5e	20	31.4	100	25	15	ni^a^	57.3	>1000
	40	49.9	200	50	10			
	80	85.5	400	75	18			
5f	20	22.2	100	50	20	87	60.2	>1000
	40	58.7	200	70	10			
	80	77.1	400	100	40			
5g	20	35.5	100	60	05	60	65.5	>1000
	40	45.5	200	80	15			
	80	60.1	400	100	15			
5h	20	16.6	100	50	15	70	76.2	>1000
	40	48.9	200	70	20			
	80	64.1	400	90	25			
5i	20	18.5	100	25	50	70	75.5	>1000
	40	46.4	200	40	20			
	80	40.5	400	50	75			
5j	20	20.5	100	30	20	ni^a^	70.1	>1000
	40	35.5	200	55	25			
	80	50.5	400	90	45			
Aspirin	20	30.4	100	30	70	60	98.3	-
	40	35.5	200	60	90			
	80	59.6	400	90	80			
Phenyl butazone	20	31.6	100	30	40	70	-	-
	40	35.5	200	60	50			
	80	57.2	400	90	80			
Control	20	-	30	-	-	ni^a^	-	-
	40		60					
	80		90					

a ni, no inhibition.

### Ulcerogenic activity

Some of the compounds in 5a-j series exhibited low ulcer production activity compared to standard drug, aspirin and phenylbutazone (10 to 25%) at 200 mg/kg po. Compounds 5a, 5b 5e and 5f have shown low ulcer production activity at 200 mg/kg po. Compound 5j elicited maximum ulcer production activity. On the contrary compound 5c, 5d 5h and 5i have exhibited ulcer production activity higher than compounds 5a, 5b, 5e, 5f and 5g but lower than compound 5j. Further compound 5g has exhibited ulcer production activity less than compounds 5c, 5d, 5h, 5i and 5j.

### Cyclooxygenase activity

Compounds 5a-d and 5f-i showed good cyclooxygenase activity on the other hand compounds 5e and 5j did not inhibit the cyclooxygenase activity.

## DISCUSSION

In 5a-j series the compound 5f with bromo group at the ortho position in ring A of benzophenone moiety, elicited maximum inhibition of oedema (58.7%). Whereas compound 5d with a methyl group at meta position in ring A of benzophenone moiety, elicited minimum inhibition of oedema (29.5%). Compound 5a with methoxy group at para position in ring A of benzophenone moiety has shown second highest anti-inflammatory activity (55.5%). Besides compounds 5h (48.9%) and 5b (48.2%) with a fluoro group and methyl group respectively at para position in ring A of benzophenone moiety have shown almost same anti-inflammatory activity. On the other hand compound 5e (49.9%) with a chloro group at meta position in ring A of benzophenone moiety has exhibited anti-inflammatory activity little higher than compounds 5h and 5b. Nevertheless compound 5i (46.4%) with two chloro groups at ortho and para position in ring A of benzophenone moiety has exhibited slightly more anti-inflammatory activity compared to the compound 5g (45.5%) having bromo group at para position in ring A. The remaining compounds 5c and 5j with a methyl group at ortho position and with no substituent in ring A of benzophenone moiety have exhibited inhibition in oedema as 39.4 and 35.5% respectively.

Compounds 5a, 5b, 5e, 5f and 5h were studied in detail at three graded doses and have shown dose dependent activity. Anti-inflammatory activity of compounds 5a-j and their comparison with standard drugs, aspirin and phenylbutazone are given in table 1.

### Ulcerogenic activity

Some of the compounds in 5a-j series exhibited low ulcer production activity compared to standard drug, aspirin and phenylbutazone (10 to 25%) at 200 mg/kg po. Compounds 5a with a methoxy group, 5b with a methyl group and 5f with a bromo group at para position and compound 5e with a chloro group at meta position in ring A of benzophenone moiety have shown low ulcer production activity at 200 mg/kg po. Compound 5j with no substituent in ring A of benzophenone moiety elicited maximum ulcer production activity. On the contrary compound 5c with a methyl group at ortho, 5d with a methyl group at meta, 5h with a fluoro group at para and 5i with two chloro groups at ortho and para positions in ring A of benzophenone moiety have exhibited ulcer production activity higher than compounds 5a, 5b, 5e, 5f and 5g but lower than compound 5j. Further compound 5g with a bromo group at para position in ring A of benzophenone moiety has exhibited ulcer production activity less than compounds 5c, 5d, 5h, 5i and 5j.

### Cyclooxygenase activity

Compounds 5a-d and 5f-i showed good cyclooxygenase activity indicating that these compounds reduce inflammatory response by inhibition of Prostaglandins. The other compounds 5e and 5j which did not inhibit the cyclooxygenase activity, therefore seems to act through some other mechanism rather than inhibiting prostaglandin synthesis.

### ALD50 studies

The toxicity study of these compounds indicates their good safety margin.

## CONCLUSION

The pharmacological activity result concludes that integration of morpholine ring into the benzophenone moiety is fruitful as most of the compounds ie., 5a, 5b, 5e, 5f and 5h were found to show potent anti-inflammatory activity. Surprisingly, compounds 5a and 5b with electron releasing groups at para position and compounds 5e, 5f and 5h with electron withdrawing groups like chloro at meta, bromo at ortho and fluoro at para position exhibits potent activity. In addition compounds 5a, 5b, 5f and 5e also show decreased ulcer production activity. Compounds 5e and 5j were found to have no suppressive effect on cyclooxygenase, which is the prime mechanism of anti-inflammatory activity.
